# Fetal Mesenchymal Stromal Cells: an Opportunity for Prenatal Cellular Therapy

**DOI:** 10.1007/s40778-018-0118-8

**Published:** 2018-02-15

**Authors:** Rachel Sagar, Lilian Walther-Jallow, Anna L. David, Cecilia Götherström, Magnus Westgren

**Affiliations:** 1 Institute for Women’s Health, University College London, 86-96 Chenies Mews, London, WC1E 6HX UK; 2 Department of Clinical Science, Intervention and Technology, K57, Division of Obstetrics and Gynecology, Karolinska University Hospital Huddinge, Karolinska Institutet, 141 86 Stockholm, Sweden; 3 Katholieke Universiteit Leuven, Oude Markt 13, 3000 Leuven, Belgium

**Keywords:** Mesenchymal stromal cells, Prenatal therapy, In utero stem cell transplantation

## Abstract

**Purpose of Review:**

The aim of the study is to provide an overview on the possibility of treating congenital disorders prenatally with mesenchymal stromal cells (MSCs).

**Recent Findings:**

MSCs have multilineage potential and a low immunogenic profile and are immunomodulatory and more easy to expand in culture. Their ability to migrate, engraft and differentiate, or act via a paracrine effect on target tissues makes MSCs candidates for clinical therapies. Fetal and extra-fetal MSCs offer higher therapeutic potential compared to MSCs derived from adult sources.

**Summary:**

MSCs may be safely transplanted prenatally via ultrasound-guided injection into the umbilical cord. Due to these characteristics, fetal MSCs are of great interest in the field of in utero stem cell transplantation for treatment of congenital disease.

## Introduction

The field of regenerative medicine has long been interested in the restorative potential of cellular therapy. Mesenchymal stem cells (MSCs) are amongst the most promising and widely studied cell sources for clinical therapy. The suggestion that there existed a subset of cells within the bone marrow which were capable of osteogenic differentiation was first published by Friedenstein et al., the early pioneer of stem cell research, as early as 1966 [1]. Forty years later, in 2006, the Mesenchymal and Tissue Stem Cell Committee of the International Society for Cellular Therapy proposed minimal criteria to define human MSCs. They must demonstrate plastic adherence; must be capable of trilineage differentiation; must express CD105, CD73, and CD90; and must lack expression of haematopoietic surface markers [2].

MSCs are, next to haematopoietic stem cells, numerically the most investigated cell type in clinical trials; as of 2015, there were 374 registered clinical trials in the NIH clinical trial database using MSCs, a threefold expansion of trials over the number noted in 2011 [3, 4]. Reflecting the large amount of pre-clinical research, ‘Prochymal’ became the first clinically licensed MSC product in 2012 when it was accepted by Health Canada to be used in severe cases of steroid-resistant pediatric graft versus host disease [5].

Clinical attention is now focused on the potential of MSCs to treat congenital disease prenatally. Osteogenesis imperfecta, otherwise known as brittle bone disease, is one such condition which shows promise for prenatal MSC treatment. Here, we discuss the features of MSCs which make them particularly suitable for cellular therapy, before describing the advantages of fetal over adult stem cell sources. We summarise current clinical experience with fetal MSCs of fetal liver origin in prenatal cellular therapy for osteogenesis imperfecta and discuss future prospects for the field.

## MSCs as a Source for Cellular Therapy

A key feature of MSCs which makes them particularly suitable for cellular therapy is their ability to migrate towards the site of injury and engraft into target tissues. It has been reported, for example, that following a fracture, MSCs may be involved in the repair of the injured bone [6]. This intrinsic response can be exploited to deliver donor cells directly to the site of injury even when the cells are infused systemically. However, they may also escape from circulation. The interaction between stromal cell-derived factor 1 (SDF-1), which is released from damaged tissues, and C-X-C chemokine receptor type 4 (CXCR4), a chemokine receptor expressed by MSCs, seems to be vital to this recruitment mechanism [7–9]. Recognition of SDF-1 by CXCR4 causes a cascade of cytoskeletal reorganisation within the MSCs, leading to cell migration towards the SDF-1 gradient. Indeed, CXCR4-negative cells are unable to home to the bone following fracture, suggesting that the migration of systemically transplanted MSCs is dependent on the presence of CXCR4 [6, 10]. Following migration, MSCs must then be able to act upon their target site. One rationale behind stem cell therapy is that cells may engraft and differentiate, thus repopulating the damaged organ with healthy cells. MSCs have been shown to home to and survive in sites of damage, but reported levels are always under 10%, decreasing with time. Surviving cells, both fetal and adult-derived, are capable of trilineage differentiation into osteoblasts, chondrocytes, and adipocytes [2].

An alternative reason for the beneficial effect of MSC transplantation is that these stem cells may additionally repair tissue by acting in a paracrine manner. The ‘MSC secretome’ consists of a wide range of factors such as cytokines, chemokines, and growth factors released by MSCs [11]. These factors are capable of modifying the microenvironment, stimulating endogenous cell proliferation, and preventing apoptosis of resident cells [12]. MSCs also release large numbers of extracellular vesicles and membrane-derived bodies containing peptides, lipids, and microRNAs that are involved in cell-to-cell communication [13, 14, 15•]. These cell communication effects could explain the therapeutic benefits that are seen even when levels of detectable MSC-derived cells are very low after infusion [16–19]. If this effect relates to stem cells or the entire cell population is today unknown [20•].

MSCs have been described as ‘medicinal signalling cells’, which secrete immunomodulatory, anti-apoptotic, anti-inflammatory, proangiogenic, promitogenic, and antibacterial factors [21]. MSCs are able to directly inhibit the proliferation of natural killer and cytotoxic T cells [22]. Specifically, MSCs increase regulatory T cells, indirectly decreasing the activity of cytotoxic T cells [23]. Thus, not only are MSCs non-immunogenic, they are also immunomodulatory.

Finally, unlike their haematopoietic and embryological counterparts, MSCs are a safe cell type for transplantation, with no significant concerns reported over 15 years of clinical use [18, 24–28, 29••]. MSCs, mainly adult in origin, have been tested in clinical trials for a diverse variety of disorders ranging from diabetes to graft versus host disease. Thousands of patients have received treatment via these trials, and few adverse events have been reported [30••]. Most importantly, there have been no reports of malignant transformation or teratoma formation in vivo. The transient nature of transplanted MSCs may be considered a positive in this regard [3, 31].

## Advantages of Fetal Versus Adult-Derived MSCs

When compared with fetal MSCs, adult MSCs, which are usually derived from the adherent fraction of bone marrow, have decreased proliferative capacity [32–34], loss of ‘fitness’ [35], and decreased anti-inflammatory capacity and homing ability [36]. However, it cannot be assumed that the earlier stem cells are obtained the ‘better’ they are, as embryonic stem cells (ESCs) derived from the inner cell mass of the blastocyst are associated with not only ethical concerns, but also those of safety. ESCs are pluripotent and must be directed towards their differentiation pathway to the desired cell lineage in order to prevent teratoma formation. Recent attention has therefore focused on fetal sources of MSCs, as they do not give rise to the pluripotency-associated issues of ESCs and yet still offer greater utility in cellular therapy than adult-derived MSCs [33, 34, 37, 38].

MSCs, whilst only constituting a minor fraction of the stem cell population, are proportionally much more prevalent during fetal life than adulthood. In the second trimester, MSCs comprise 1:3000 blood cells and 1:400 bone marrow cells [37, 39], whilst they make up 1:10,000 bone marrow cells in a newborn and only 1:2 × 10^6^ in an 80-year-old [40]. Fetal MSCs are therefore found at higher frequency in tissues than adult MSCs and can be readily obtained from numerous sources of both fetal and extra-fetal tissues. These comprise fetal liver, umbilical cord, umbilical cord blood, amnion, placenta, and amniotic fluid [41–43]. Amniotic fluid MSCs can be acquired from 15 weeks of gestation, either through amniocentesis, amniodrainage, or late during gestation at caesarean section. Amniotic fluid contains fetal cells from the amnion, skin, and respiratory system that can differentiate into MSCs or HSCs [44]. Placental stem cells can be procured from chorionic villus sampling as early as 11 weeks of gestation or at delivery from the placenta. Placenta contains a large variety of cells including MSCs [44]. Umbilical cord blood stem cells can be obtained from cordocentesis during pregnancy or at delivery, and the umbilical cord and amnion itself are readily available and frequently discarded, following delivery. Fetal liver MSC, and all of the fetal-derived MSC listed above, can also be obtained at termination of pregnancy.

Not only are fetal MSCs easily obtainable in relatively high concentrations, but they also offer practical advantages over their adult counterparts. First-trimester fetal MSCs attain four times as many population doublings in 50 days compared to adult MSC: 28.4 versus 7.1, a consequence of their much shorter doubling time (30 versus 80 h) [33, 34, 37, 45–47]. This extensive proliferative capacity is also increased by the ability of fetal MSCs to undergo more population doublings than adult MSCs, reaching senescence after 70 doublings versus 15 to 40 in adult cells [33, 34, 48–50]. Fetal MSCs also have about two times higher colony-forming unit-fibroblast capacity compared to adult MSCs [34]. Fetal MSCs hence offer advantages with respect to the practicalities of cell culture and feasibility for clinical use. Caution needs to be executed when evaluating these data since extensive proliferation is not equivalent to self-renewal.

In addition to being, practically speaking, a better source of stem cells, fetal MSCs may also be more effective in avoiding immune recognition. Fetal MSCs are widely held to be less immunogenic than their adult counterparts [45, 51–53]. Fetal MSCs express lower surface levels of HLA class I than adult cells [34, 45, 51]. Fetal MSCs do not express cell surface or intracellular HLA class II, whilst untreated adult MSCs are positive for the intracellular form. When stimulated with IFN gamma, surface expression of HLA class II is induced after only 1 day in adult MSCs and after 7 days in fetal MSCs [22, 51]. It therefore appears that fetal MSCs are, at least initially, less immunogenic than adult MSCs.

Finally, fetal MSCs may also be more suitable for clinical treatment given their increased ‘fitness’ as stem cells. It has been reported that MSCs shift their differentiation commitment from the osteogenic to the adipogenic lineages with increasing cellular senescence [54], and it may be that fresh (non-senescent) fetal MSCs have superior osteogenic therapeutic potential to that of adult MSCs. Guillot et al. compared the basal expression of osteogenic genes in first-trimester liver, blood, and bone marrow MSCs to adult bone marrow MSCs and found that fetal MSCs had higher levels of expression of all 16 osteogenic genes [55]. When differentiated down the osteogenic pathway, fetal MSCs reached higher levels of osteogenic gene upregulation than adult MSCs. Similarly, Zhang et al. found that this translated into superior osteogenic capacity, with higher levels of calcium deposited and higher alkaline phosphatase activity [34]. Their superior differentiation capacity does not only apply to the osteogenic lineage. In addition to differentiation down the osteo-, chondro-, and adipogenic lineages, fetal MSCs can also differentiate into muscle cells (myoblasts) and oligodendrocytes [27, 34, 38, 48, 56–60].

## Benefits of Prenatal Treatment

There are also many potential advantages to offering stem cell treatment at the prenatal stage. Many congenital conditions are now diagnosed on the mid-trimester anomaly ultrasound scan, and increasingly, non-invasive prenatal diagnosis is becoming available even earlier for disorders such as achondroplasia and atophoric dysplasia [61]. However, there are few prenatal treatments available. Psychosocially, this can be a very difficult time for parents, knowing their child has a disability, with their only options to ‘wait and see’ how their child is at birth, wondering if their condition is worsening whilst in utero. Treating a child affected by a congenital disease before birth offers an improved situation for the parents of the child, to feel that they are ‘doing something’.

Physiologically, there are many potential advantages to in utero treatment. Treating early, before a disease has the opportunity to have an effect on a fetus, will result in a better condition at birth. Furthermore, the average weight of a 20-week fetus, at 300 g, is over ten times smaller than that of the average newborn baby. There is therefore the opportunity for greatly increased cell dosage given the size and weight of the fetus compared with the neonate. Additionally, the physiological conditions for systemic distribution of the MSC are better in the fetus than in the neonate or adult because the fetal circulatory shunts such as the foramen ovale and ductus arteriosus reduce distribution of the MSCs to the pulmonary circulation, avoiding their sequestration in the lungs as occurs after birth [62, 63•, 64, 65]. As described above, fetal life is a time of stem cell proliferation and migration to different anatomic compartments; thus, the transplanted stem cells will migrate alongside affected ones. Higher levels of engraftment during fetal life have also been reported, especially when a fetal-to-fetal transplantation approach is applied [66]. Finally, the relative naiveté of the immune system results in less rejection, no need for myeloablation, and permits the development of immune tolerance towards donor cells [67–70].

## Osteogenesis Imperfecta

Osteogenesis imperfecta (OI) is a disorder of type 1 collagen with a prevalence of 1/20,000 that is commonly diagnosed prenatally. Clinically, affected individuals experience abnormal skeletal development, osteopenia, multiple painful fractures, and short stature. Ninety percent of cases are autosomal dominant, due to mutations in COL1A1 or COL1A2, the genes encoding the subunits of type 1 collagen. The Sillence classification published in 1978 described four types of OI, ranging from mild type 1 to the progressively deforming types 3 and 4 and to the perinatally lethal type 2 [71]. More recently, up to 15 other types have been described which affect genes controlling modification and processing of type 1 collagen, often recessive in inheritance [72]. Current postnatal treatments are non-curative and aim to decrease deformity, relieve pain, promote normal function, and improve quality of life.

Severe cases of OI are suspected on ultrasound due to abnormal fetal growth, fractures, and deformity or bowing of limbs seen in mid-gestation. Definitive diagnosis requires mutation analysis on a chorionic villus sampling, amniocentesis, or fetal blood sample and is reached using next-generation sequencing techniques. However, currently, no antenatal treatment options are available.

A number of pre-clinical studies suggest that in mouse models of OI, MSCs transplanted in utero or in early neonatal life equivalent to a late pregnancy human fetus are capable of migration to the bone, engraftment, and differentiation to osteoblasts, where they improve bone mechanical properties and the clinical phenotype [44, 47, 55, 73–77].

OI is diagnosed in utero, there are no current curative treatments, and mouse models provide evidence of efficacy of treatment. Therefore, OI is a condition which is amenable to prenatal MSC therapy. Here, we summarise the clinical experience of prenatal cellular therapy with fetal-derived MSCs for OI.

## Clinical Experience with Fetal MSCs in Prenatal Cellular Therapy for OI

Two case studies of prenatal transplantation of allogenic human first-trimester liver-derived MSCs in type III and type IV OI patients have been published [28, 29]. They were performed at the Karolinska Institutet in Sweden and at the National University Hospital in Singapore. Whilst it is difficult to definitively determine the effect of prenatal fetal MSC transplantation in these two heterogeneous cases, the results suggest that it is safe and a potential clinical benefit, particularly in comparison to an untreated Canadian patient with the same mutations as patient A who succumbed at 5 months of age.

### Patient A

Patient A, who has severe type III OI with intrauterine fractures, received an infusion of 5.0 × 10^6^ cells/kg body weight via the umbilical vein (total dose, 6.5 × 10^6^ cells with a viability of 90%) of first-trimester liver-derived MSCs at 31 weeks of gestational age. No signs of fetal distress were observed during the injection. The pregnancy was uncomplicated after the infusion, but a spontaneous preterm rupture occurred at 35 weeks of gestational age, and patient A was delivered by caesarean section.

Alloreactivity against donor MSCs was not detected pre-transplantation or at 9 months or 6 years of age, and the patient’s immunocompetency was confirmed at all these time points.

Evidence of osteoblastic differentiation of donor MSCs was detected in a bone biopsy specimen taken at 9 months of age. This biopsy also showed regularly arranged and configured bone trabeculae lined by a columnar layer of normal osteoblasts, normal amount and distribution of osteocytes and ossification, and no apparent signs of healing or remodelling. Patient A was started on bisphosphonate therapy from 4 months of age due to vertebral compression fractures.

Patient A’s clinical course was better than expected from her genetic mutation for the first 2 years. She experienced only two clinically suspected fractures (a clavicular fracture at 6 weeks and a costal fracture at 9 months), and at 15 months, a fall from 1 m resulted in a femoral fracture that healed rapidly after initial stabilisation with a bandage. She grew along her own growth velocity curve at − 5 standard deviations (SD) for height and weight. However, from 6 to 8 years of age, the lengthwise growth decreased and the fracture frequency increased, with some prolonged fractures healing. Thus, the decision was made to administer a second transplant.

Patient A was administered an intravenous infusion of 2.8 × 10^6^ cells/kg body weight (total dose, 42 × 10^6^ cells) from the same fetal donor as the prenatal infusion at 8 years and 2 months of age. No adverse events (AEs) were noted. Despite a low level of engraftment measured at 8 years and11 months, over the next 2 years, she experienced no new fractures (compared to the previous two fractures per year), linear growth increasing from the − 6.5 SD to the − 6 SD curve, and decreasing bone mineralisation. Her ability to walk improved to 1000 m without difficulty, and she started dance classes, increased participation in gymnastics, and played modified indoor hockey. She subsequently received booster doses from the same donor at 10, 12, and 13 years of age, respectively. The absence of new fractures and improved growth velocity continue.

### Patient B

Patient B, who had type IV OI with intrauterine fractures, received a prenatal infusion via the hepatic vein of ~ 30 × 10^6^ cells/kg body weight (total dose, 40 × 10^6^ cells) of first-trimester liver-derived MSCs at 31 weeks of gestational age. No signs of fetal distress were observed through 1 h post injection. No new fractures were noted in the following 7 weeks, and the child was delivered by elective caesarean section at 38 weeks + 3 days of gestational age.

Alloreactivity against donor MSCs was not detected pre-transplantation or at 7 weeks post transplantation, and the patient’s immunocompetency was confirmed at both these time points.

Patient B was started on bisphosphonate therapy from 1 month of age because of poor mineralisation; her mineralisation increased from a neonatal *Z* score of − 7.3 to − 0.9 at 13 months of age. No new fractures were detected in the first year of life. The patient followed her own growth curve just below the third percentile until 1 year of age, when the longitudinal length plateaued. Thus, a booster dose of 10 × 10^6^ cells/kg (total dose, 88 × 10^6^ cells) from the same fetal donor was administered by intravenous infusion at 19 months of age. No AEs were observed. Her growth subsequently continued at just below the third percentile, and she started to walk shortly after the transplantation.

The Karolinska Institutet has treated two other children suffering from intrauterine fractures and who were diagnosed with type III OI prenatally, but has not yet published the cases. The first patient was administered 10.3 × 10^6^ human first-trimester fetal liver-derived MSCs/kg at gestational age of 28 weeks + 3 days in 2015, and the second patient was administered 2.3 × 10^6^ human first-trimester fetal liver-derived MSCs/kg at corrected age of 38 weeks + 2 days. Neither patient experienced any AEs. Neither patient experienced fractures after birth (up to 1 year and 2 months of age for the first patient and 1 year for the second patient).

The National University Hospital in Singapore treated one other OI patient with a prenatal infusion of 50 × 10^6^ cells/kg allogenic human first-trimester fetal liver-derived MSCs at gestational age 32 weeks + 1 day in 2014. The patient experienced no AEs and no fractures after birth (up to 1 year of age).

We are also aware of two type III OI patients with intrauterine fractures treated with haploidentical adult bone marrow-derived MSCs (one dosed at 2 × 10^6^ cells/kg body weight and one dosed at 3 × 10^6^ cells/kg) at 31 weeks of gestational age in Chile in 2005 and 2006 (personal communication). No engraftment was seen (analysed in collaboration with the Karolinska Institutet), but the two patients had no fractures and normal growth after birth and no AEs. Long-term status is unknown. Two type III OI patients with intrauterine fractures have been treated with haploidentical adult bone marrow-derived MSCs in Italy, but no further information is available.

## Conclusions and Future Perspectives

Fetal stem cells have shown promise in reducing fracture rates in a mouse model [49, 50, 77, 78], and engraftment of allogeneic cells in human fetuses with OI has also been shown [28, 29]. As discussed above, a few patients with OI have been treated with fetal MSCs before and/or after birth with promising results. So far, however, the MSC treatment for OI and other diseases has been performed on a case-by-case basis. Carefully planned and ethically and regulatory approved clinical trials are needed to advance the field and to determine whether these encouraging initial findings indeed show that prenatal therapy in the end will benefit the patients.

One such study is the BOOSTB4 (Boost Brittle Bones Before Birth) study that is to be initiated shortly. BOOSTB4 is a European multicenter phase I/II study with the overall aim to develop the advanced therapy medicinal product (ATMP) fetal MSC. In BOOSTB4, the safety, clinical effectiveness, acceptability, and cost-effectiveness of prenatal and postnatal transplantation or postnatal transplantation only of fetal-derived MSCs to treat severe but not lethal forms of OI will be evaluated.

OI serves as a useful disease model to explore the acceptability, safety, and efficacy of prenatal transplantation of fetal MSCs. Successful clinical demonstration of BOOSTB4 cell therapy in OI will hopefully build knowledge and experience for the design of clinical trials for the treatment of other developmental fetal disorders that could be amenable to prenatal MSC transplantation. These include other skeletal dysplasias, muscular dystrophies, and inborn errors of metabolism with early central nervous system involvement that are increasingly diagnosed in utero.

To be noted is that ethics approval and approval from national or regional regulatory authorities are mandatory when performing any clinical research or trials. It is important to follow directives and regulations to develop approved processes that comply with GMP when manufacturing ATMP products. Any AEs, side effects, and long-term safety risks must be assessed and mitigated, since transplanted cells may remain for many years in patients’ bodies. Therefore, careful monitoring and extended follow-up of patients who receive pre- and postnatal stem cell treatments are vital.
